# Acceptability, feasibility and cost of point of care testing for sexually transmitted infections among South African adolescents where syndromic management is standard of care

**DOI:** 10.1186/s12913-023-10068-8

**Published:** 2023-10-10

**Authors:** Rebecca Marcus, Pike C, K. Gill, P. Smith, S. Rouhani, A. Mendelsohn, E. Mendel, N. Lince-Deroche, K. Naidoo, N. Ahmed, O. Stirrup, J. Roseleur, R. Leuner, G. Meyer-Rath, L. G. Bekker

**Affiliations:** 1 Desmond Tutu HIV Centre, University of Cape Town, Cape Town, South Africa; 2 North Middlesex University Hospital, London, UK; 3 Health Economics and Epidemiology Research Office, University of the Witwatersrand, Johannesburg, South Africa; 4 Mortimer Market Centre, Central North West London NHS Trust, London, UK; 5 Institute for Global Health, University College London, London, UK; 6 Department of Global Health, School of Public Health, Boston University, Massachusetts, USA

**Keywords:** Adolescents, STIs, South Africa, Costs

## Abstract

**Background:**

Young people (YP) in southern Africa are at substantial risk of HIV and sexually transmitted infections (STIs). Despite the epidemiological and biological link between STIs and HIV transmission and acquisition, infections such as *Chlamydia trachomatis* (CT) and *Neisseria gonorrhoeae* (NG) remain widely undiagnosed. Syndromic STI management is the standard of care in low- and middle-income countries (LMICs) despite a high prevalence of asymptomatic infections. We conducted an observational study to explore the acceptability, feasibility, and cost of a STI test-and-treat service for YP in Cape Town.

**Methods:**

YP attending a mobile clinic (MC) and a youth centre clinic (YC) were offered STI screening. Urine testing for CT and NG using a 90-min molecular point-of-care (POC) test on the GeneXpert platform was conducted and treatment provided. Data were collated on demographics, sexual behaviour, presence of symptoms, uptake of same-day treatment, prevalence of CT/NG, and service acceptability.

**Results:**

Three hundred sixty six participants were enrolled (median age 20, 83% female).57% (209/366) of participants tested positive for either CT (126/366, 34%) or NG (57/366, 16%) or co-infection (26/366, 7%).

Clinical symptoms were a poor predictor of GeneXpert diagnosed CT or NG, with a sensitivity of 46.8% and 54.0% for CT and NG respectively. Although half of participants initially chose to receive same day results and treatment, only a third waited for results on the day. The majority of participants (91%) rated the service highly via a post-visit acceptability questionnaire.

**Conclusion:**

Curable STIs are highly prevalent in this population. STI screening using POC testing was feasible and acceptability was high. The study provides further impetus for moving policy beyond syndromic management of STIs in South Africa.

**Supplementary Information:**

The online version contains supplementary material available at 10.1186/s12913-023-10068-8.

## Background

The World Health Organization (WHO) identified the scale-up of interventions to tackle sexually transmitted infections (STIs) as a priority in their Global Health Sector Strategy for HIV, viral hepatitis and STIs 2016–2021 [1], highlighting the importance of providing STI prevention and care in primary health, sexual and reproductive health (SRH), and HIV services, with the goal of ending STI epidemics as a major public health concern by 2030.

The high prevalence of curable STIs amongst young people in sub-Saharan Africa (SSA), particularly young women, has been well described [2–5]. A meta-analysis of 18 HIV prevention studies in SSA found *Chlamydia trachomatis* (CT) and *Neisseria gonorrhoea* (NG) prevalence ranging between 10–15% and 1.7–4.6% respectively in primary healthcare populations aged 15–24, with CT/NG prevalence particularly high amongst South African (SA) participants [6]. STI prevalence is higher still in some SA provinces, with over half of participants in an adolescent oral Pre-Exposure Prophylaxis (PrEP) study in Cape Town and Johannesburg testing positive for at least one STI at baseline [5]. The majority diagnosed with CT/NG were asymptomatic, further highlighting the poor sensitivity of syndromic STI management. 

The effect of untreated STIs is substantial, both as a co-factor for HIV transmission, and for short- and long-term reproductive sequelae for women and men [7, 8]. Adolescents and young people are specifically vulnerable to both STIs and HIV [9], underlining the necessity for adolescent-responsive STI services. STIs in low-resource settings are largely managed syndromically, relying on patient-reported symptoms to provide treatment for a number of potential infections. While this approach offers an immediate and relatively low-cost pathway in resource-limited, high HIV burden settings, it is inadequate in treating and preventing STIs due to its inability to detect asymptomatic infections, leading to both over and under treatment of STIs and adding to the risk of antibiotic resistance [10–12].

WHO guidance recommends moving towards aetiological diagnosis over syndromic management of STIs. Affordable and feasible diagnostic options are urgently required, this may include point-of-care (POC) diagnostics [1]. The integration of test and treat programmes into community-based HIV/SRH services has the potential to reduce STI-related morbidity and further STI and HIV transmission in communities where HIV is prevalent. In 2011, SA rolled out POC testing for tuberculosis (TB) in primary care clinics using GeneXpert (Cepheid, Sunnyvale, CA, USA), where they request patients to return within 1–2 days for results [13]. The same technology can be used to test for CT/NG, providing rapid diagnosis and increasing the potential for same day treatment. Although GeneXpert machines are generally located in nearby laboratories, their widespread availability presents an opportunity for STI management in SA. There are limited data evaluating POC STI testing in adolescent populations, a population at risk of both STI and HIV infection in South Africa [5]. The primary aim of this study was to evaluate the feasibility and acceptability of integration of POC STI testing into health services for young people (15–23 years) in low-income, high-burden environments. To inform implementation, a secondary aim was to assess the cost of offering POC STI testing versus the standard syndromic approach.

## Methods

### Study design

This prospective study evaluated a POC STI screening and treatment programme for 15–23 year-olds using adolescent-responsive health services in Cape Town, SA.

### Study setting and population

The study was conducted in 2017 at two sites, the first a clinic in a township youth centre (YC) in the southern peninsula of Cape Town, and the second, a mobile clinic (MC) that parks on rotation at different low-income, high-density locations in the Cape Town area. The MC follows a set rotation schedule, serving a different location each day. Participants are made aware of the rotation schedule at study visits and via social media. Both clinics are established and provide free healthcare for adolescents and young people (aged 12–23 years), including HIV and pregnancy testing, oral PrEP, STI syndromic management, and SRH services. The mobile clinic is staffed by three counsellors and a nurse, and the youth centre clinic staffed by one doctor, one nurse and two counsellors.

Young people attending the YC and MC for a health check were invited to participate in a STI test-and-treat programme for CT and NG if eligible. Posters displayed at the clinics provided information about the programme, with no other outreach recruitment or advertising. Eligible participants were sexually active (reporting sexual activity in the past two years), 15–23 years old, and able to provide signed informed consent. Those who had recently been treated for a STI or presumed STI were invited to return after 3 weeks. Enrolment was voluntary and no reimbursement was offered for participation.

### Ethical considerations

Ethical approval was granted by the Human Research Ethics Committee (HREC) at the University of Cape Town’s (UCT) Faculty of Health Science (Ref 339/2016). The need for parental informed consent was waived by the UCT HREC on that basis that young people access these health services routinely without need for parental consent and obtaining parental consent may have presented a barrier to participation. All individuals therefore gave independent voluntary informed consent regardless of age. The YC adolescent and adult community advisory board (CAB) reviewed and supported the study. All procedures were performed in accordance with the Declaration of Helsinki.

### Study procedures

Written informed consent was sought from all participants prior to enrolment. Consent forms and study documentation were translated into local languages (isiXhosa and Afrikaans) and administered in the participant's preferred language. Trained clinic staff administered a sexual risk behaviour and symptom questionnaire and collected sociodemographic data.

Clinic staff asked participants about symptoms as per the syndromic management approach, then offered STI screening and treatment with a single urine sample for CT/NG using the NAAT (Nucleic Acid Amplification Test)-based GeneXpert® CT/NG assay (Cepheid, Sunnyvale, CA, USA) on a 4 module configuration. Clinical staff were trained on the GeneXpert® platform, which allows dual detection of CT/NG in 90 min. High sensitivity and specificity (> 95%) has been demonstrated for CT/NG using endocervical, vaginal and urine samples [14], with urine samples used in preference to vaginal samples for women due to the ease of sample collection. Clinical examination was performed by nursing or medical staff if clinically indicated.

HIV testing, risk reduction counselling, condoms and other methods of contraception were offered. A paper-based acceptability questionnaire in the patient’s preferred language was adapted from a pre-existing tool derived from common desirable aspects of adolescent youth friendly healthcare services [15, 16]. Participants were asked to rate the STI testing programme using five point Likert scale questions.

### Test results and treatment

YC participants could opt to wait for test results and receive treatment on the same day or return the following day. MC participants were asked to wait for results, as the clinic frequently rotated its location. If MC participants were unable to wait however, they could choose to receive results by phone (including text message), or to return to the MC at a scheduled location. Those with positive results who did not return for treatment were contacted by phone or text message where possible. Those diagnosed with CT or NG were offered immediate treatment with antibiotic therapy compatible with national guidelines [17]. Those with vulval symptoms suggestive of candidiasis were offered Clotrimazole, and those with vaginal discharge were offered treatment for bacterial vaginosis and trichomoniasis vaginalis as per national guidelines. Participants with positive CT and/or NG tests were given contact slips for partner(s) to take to a local clinic for treatment. Partners could enrol if they met the age criteria (15–23 years) to access care at the youth or mobile clinics.

### Data analysis

Analyses were conducted using Stata 14.0 (Stata Corporation LP, College Station, TX). Descriptive statistics (median [IQR] for continuous variables and n [%] for categorical variables) were used to characterise baseline distributions of study variables. Sensitivity, specificity, positive predictive and negative predictive values were calculated using the GeneXpert test results and self-reported symptoms to assess the utility of clinical symptoms in diagnosing a genital infection. Feasibility was assessed by the proportion of participants receiving same-day testing, results, and treatment if appropriate, and by acceptability ratings by participants. There was no predetermined threshold to define feasibility.

### Cost analysis

#### Scenarios

Resource use and costs for provision of syndromic management and POC testing were estimated from the provider perspective for the YC only (see Table 1). For the POC scenario, we assumed that the GeneXpert machine was used solely for CT/NG testing. In addition, a hypothetical non-POC scenario (Lab) was modelled that assumed GeneXpert testing was conducted at the nearest state-run laboratory as per the GeneXpert TB diagnostic model in South Africa.

**Table 1:** Summary of STI screening and testing scenarios

**Scenarios:**	**Syndromic management (SM)**	**Point-of-Care (POC)**	**Laboratory** (lab)
Clients served	Symp. only	Symp. & asymp	Symp. & asymp
Includes:			
Consultation	Yes	Yes	Yes
Physical exam	Yes	If symptomatic	If symptomatic
GeneXpert testing	No	Yes, on-site	Yes, lab-based
Same day treatment	Yes	Yes	No
Disease-specific treatment	No	Yes	Yes

*STI* Sexually transmitted infection, *Symp.* Symptomatic, *Asymp.* Asymptomatic

*POC* Purchase price of GeneXpert and all costs are attributed to CT/NG clients

#### Cost data collection and analysis

Clinics were visited by a specialised costing team before and during the POC intervention to cost syndromic and POC approaches. Data collection followed best practice for micro-costing, an ingredients-based, bottom up approach [18]. Personnel, supplies, equipment, medicine and space-related costs (i.e. buildings and utilities) were included. The cost of training on syndromic management or POC testing as well as patient costs were excluded. The spaces used for clinical provision were observed and measured at each visit. Staff described the clinic flow and approximate time required for each task. Staff recorded the duration and content of each consultation.

Resource lists for each observed scenario (including POC) were compiled and costs were obtained including personnel, supplies, equipment, and medicines from publicly available documents including Department of Public Service and Administration remuneration guidelines and National Department of Health procurement and tender documents. Buildings were costed using approximate rental cost per square metre [19–21]. Equipment costs were annualised linearly using the local discount rate of 6.5% (i.e. the repurchase rate set by the SA Reserve Bank in 2017) and depreciation periods recommended for various categories of equipment by the SA Revenue Service [22]. For POC, the GeneXpert purchase cost was spread across CT/NG tests conducted throughout the assumed 5-year lifespan of the machine. For Lab, costs associated with the GeneXpert machine, including shipping and storage, were assumed to be included in the per test charge levied by the laboratory (National Health Laboratory Service Pricelist 2017). All costs were collected in SA Rand (ZAR) and, if needed, were inflated to 2017 values using SA Consumer Price Index data [23]. Costs are reported here in 2017 US dollars (US$) using the average annual exchange rate for 2017 of 13.32 ZAR per 1 USD [24].

The average total cost per patient seen and managed was calculated for each scenario. For the syndromic management scenario, we present the average cost per patient seen. In the POC and Lab scenarios, we present the average costs per patient tested and treated if positive.

## Results

### Characteristics of the study population

A total of 366 participants (YC 209; MC 157), with a median age of 20 years (IQR 18–23; range 15–23) were enrolled over a nine month period. The mobile clinic visited 23 locations during the recruitment period, with recruitment occurring most week days at both sites. Demographic characteristics were similar between sites, with the majority of participants being female (83%, 303/366; Table 2) and 40% overall enrolled in education (143/366). Presence of STI symptoms was the most common reason for attending the YC clinic (48%, 100/209) and HIV testing the most common reason for attending the MC (40%, 63/157). Overall, two-thirds of participants consented to HIV testing (YC 52%, 108/209; MC 89%, 139/157), with five participants newly diagnosed with HIV infection (1%).

**Table 2:** Characteristics and sexual risk behaviours of adolescents enrolled in the programme

	**YC (*n* = 209)**		**MC (*n* = 157)**		**Overall (*n* = 366)**	
	**N**	**%**	**N**	**%**	**N**	**%**
**Gender**						
Female	169	81	134	85	303	83
Male	40	19	23	15	63	17
**Age (years)** (median (IQR))	20	18–21	20	18–23	20	18–23
**In education**						
Yes	80	38	63	40	143	39
No	120	58	86	55	206	56
Unknown	9	4	7	5	17	5
**Highest Level of Education**						
Tertiary (completed or pending)	42	20	8	5	50	14
Matriculated	60	29	69	44	129	35
Secondary school or lower	106	51	76	48	182	50
Unknown	1	0	4	3	5	1
**Employed**						
Yes	37	18	32	20	69	19
No	151	72	119	76	270	74
Unknown	21	10	6	4	27	7
**Reason for presentation**						
Contraception	33	16	8	5	41	11
HIV test	19	9	63	40	82	23
STI symptom	100	48	11	7	111	30
Multiple (HIV, STI, ± FP)	39	19	59	38	98	27
Other	18	9	11	7	29	8
Unknown	0	0	4	3	4	1
**Tested for HIV**	108	52	139	89	247	68
**Positive HIV test**	3	1	2	1	5	1
**Age at sexual debut (years)** (median (IQR))	16	15–17	16	15–17	16	15–17
**Number of partners in past 3 months**						
1	179	86	125	80	304	83
2	20	10	28	18	48	13
≥ 3	10	5	2	1	12	3
Unknown	0	0	2	1	1	1
**Condom use**						
Never	26	12	25	16	51	14
< Half of the time	20	10	33	21	53	14
≥ Half of the time	133	64	75	47	208	57
Always	30	14	23	15	53	14
Unknown	0	0	1	1	1	1
**Partner has had > 1 partner in past 3 months**						
Yes	61	29	42	27	103	28
No	66	32	36	23	102	28
Unsure/Unknown	82	39	79	50	161	44
**Partner ≥ 5 years older**						
Yes	42	20	44	28	86	23
No	162	78	99	63	261	72
Unsure/Unknown	5	2	14	9	19	5
**Perceived risk of contracting an STI**						
0%	15	7	11	7	26	7
10–30%	79	38	45	29	124	34
40–60%	70	33	54	35	124	34
≥ 70%	45	22	43	27	88	24
Unknown	0	0	4	2	4	1

The median age at sexual debut was 16 years (IQR 15–17). 16% (60/366) reported more than one sexual partner in the past three months, and almost a quarter (23%, 86/366) stated their sexual partner was at least five years older than them. Fourteen percent (53/366) reported always using condoms, 14% (51/366) never used condoms, and the remaining 72% (262/366) reported inconsistent condom use. Despite this, only 24% (88/366) perceived themselves to be at high risk of acquiring an STI. Table 2 describes baseline characteristics for the cohort.

### Prevalence of chlamydia and gonorrhoea amongst participants

50% (183/366) of participants tested positive for either CT or NG. Over a third of participants tested positive for CT (34%, 126/366) (Table 3), and 16% for NG (57/366). Co-infection with both CT and NG was found in 7% (26/366) of participants. At both sites, there was one test failure for CT and NG (device-related). Clinical symptoms were a poor predictor of GeneXpert diagnosed CT/NG, with a sensitivity of 46.8% and 54% for CT and NG, respectively (Tables 3 and 4).

**Table 3:** Diagnostic performance of self-reported symptoms compared to GeneXpert testing for prediction of *Chlamydia trachomatis* and *Neisseria gonorrhoeae* infection among adolescents enrolled in the STI test-and-treat pilot program in Cape Town, South Africa at Youth Clinic and mobile clinic study sites

	GeneXpert Determined positive	GeneXpert Determined negative	Total	
*Chlamydia trachomatis*				
Reported symptoms	59	87	146	PPV 0.4%
No reported symptoms	67	151	218	NPV 69.3%
Total	126	238		
	Sensitivity 46.8%	Specificity 63.4%		
*Neisseria gonorrhoeae*				
Reported symptoms	31	115	146	PPV 21.2%
No reported symptoms	26	192	218	NPV 88.1%
Total	57	307		
	Sensitivity 54.4%	Specificity 62.5%

**Table 4:** Average total cost per scenario (and per cost component) for *Chlamydia trachomatis* and/or *Neisseria gonorrhoeae* management (Here, management equates to “treated” for SM and "tested and treated if needed" for POC and Lab)(2017 USD)

	**Cost**	**Uncertainty range**^**a**^	**% of total**
**Scenario 1: SM – Syndromic management (Cost per case managed)**			
Personnel	10.61	(7.96–13.26)	66.3%
Supplies	0.48	(0.36–0.59)	3.0%
Equipment	1.57	(1.18–1.97)	9.8%
Laboratory	-	-	-
Medicine	0.91	(0.91–0.91)	5.7%
Overhead	2.44	(1.83–3.05)	15.2%
**Total**	**16.01**	**(12.23–19.78)**	**100.0%**
**Scenario 2: POC – Point-of-care (Cost per case managed)**			
Personnel	11.54	(8.66–14.43)	20.7%
Supplies^b^	18.73	(14.04–23.41)	33.6%
Equipment^b^	22.63	(16.97–28.29)	40.7%
Laboratory	-	-	-
Medicine	0.25	(0.25–0.25)	0.5%
Overhead	2.54	(1.91–3.18)	4.5%
**Total**	**55.70**	**(41.84–69.56)**	**100.0%**
**Scenario 3: Lab – Testing at state laboratory (Cost per case managed)**			
Personnel	10.36	(7.77–12.96)	35.9%
Supplies	0.78	(0.58–0.97)	2.7%
Equipment^c^	1.46	(1.09–1.82)	5.1%
Laboratory^c^	13.63	(10.32–17.04)	47.3%
Medicine	0.25	(0.25–0.25)	0.9%
Overhead	2.35	(1.76–2.94)	8.2%

^a^Cost ranges represent uncertainty analysis (i.e. ± 25% for staff time and supply and equipment costs). Medicine costs are not varied because they are based on published South African tender prices

^b^GeneXpert instrument costs for the POC scenario are included under equipment, and cartridge costs under supplies

^c^For the Lab scenario, the laboratory costs are the charge for testing. They include the cartridge costs and reflect the shared use of the GeneXpert instrument at the laboratory. Equipment in the Lab scenario includes other equipment such as the examination bed, desks, etc.

### Feasibility and Acceptability of POC STI testing

**Fig. 1:**
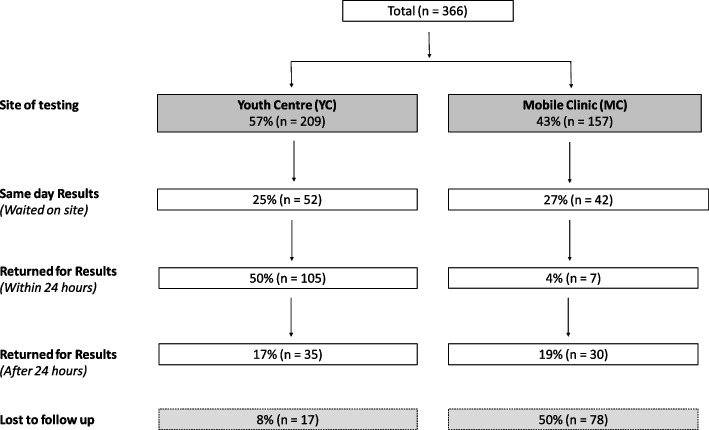
Participants retained by time point

Of 209 YC participants, the majority (66%, 138/209) initially indicated that they would like to receive their results on the same day, while the rest said they would return the following day (Fig. 1). In practice, only 25% (52/209) of the cohort waited for results, 50% (105/209) returned the following day, and a further 17% (35/209) were successfully recalled. 8% (17/209) never returned despite repeated attempted recalls via phone or text messaging. Although all 157 MC participants were asked to wait for their results, only 27% (42/157) actually waited. 4% (7/157) returned the following day and 19% (30/157) were successfully recalled. 50% (78/157) participants never returned despite follow up.

The post-test acceptability survey was self-completed with an 81% response rate (*n* = 295). Overall, the majority (91%, 267/295) of participants reported the POC testing service was helpful, a service they would use if it was available in traditional clinics (94%, 277/295), and a positive experience (80%, 237/295). 5% (14/295) of participants expressed anxiety around receiving their results, the prospect of a positive test, and potential consequences if it became known that they had tested. YC participants reported a slightly more positive experience than MC participants, with 93% of YC participants finding the service helpful versus 85% at the MC, and 91% and 83% rating the experience as good at the YC and MC respectively.

The cost per patient seen for syndromic management was least costly at $16.01. Aetiological treatment with GeneXpert cost $55.70 per patient tested on-site in the POC scenario, with off-site GeneXpert testing in the Lab scenario less expensive at $28.84 per patient tested. Personnel costs comprised two-thirds of total costs for syndromic management, whereas equipment costs, mainly GeneXpert, were two-fifths of the POC scenario cost.

## Discussion

This pilot study demonstrated acceptability of POC STI testing and treatment among youth attending a youth centre and mobile SRH service in Cape Town, and the feasibility of same day testing. Participation was voluntary and without reimbursement, indicating that the positive feedback received was likely due to strong support for the service among users.

The prevalence of CT and NG amongst participants was high (50%), aligning with data from previous studies in this population [6, 25–27]. Using POC diagnostics, STIs that would have gone undetected and untreated using syndromic algorithms were diagnosed. The potential sequelae of these untreated genital infections underline the importance of offering STI testing and treatment to populations at risk in high-burden settings. It is possible that STI testing as part of a SRH service for adolescents could present a valuable drawcard to encourage health-seeking behaviours such as HIV testing and prevention opportunities [28].

The proportion of adolescents willing to wait for results is an important factor to consider when designing STI testing interventions and modelling their effectiveness as adolescents often describe waiting times as one of the barriers to engaging in health services [8, 15, 25]. Despite the provision of WiFi, computer access and areas to socialise and play sports, only 25% of YC participants waited the full 90 min to receive their results. At the MC, where there was little provided to occupy the participants but where they were actively requested to stay, a similar proportion waited (27%) overall. In both settings, the wait was too long to retain a majority of adolescent participants.

A more notable difference was the extent of return, where the majority of YC participants returned and received their results (83%) compared to only 50% of MC participants. This is likely due to the fixed-site nature of the YC in the community, whereas the MC changed locations regularly, making return less convenient or possible.

Although same-day testing in this population was feasible, the difficulties providing contemporaneous in-person results and treatment highlights the pragmatic challenges associated with providing a same day service, which is of greater relevance when providing mobile versus fixed-site health services. These findings support the need for shorter waiting times or the possibility of results communicated by phone or text message, and a script for treatment potentially collected at a fixed venue or couriered. The latter option has shown success amongst men who have sex with men in the United Kingdom [29, 30], but is yet to be evaluated amongst youth in LMICs. There is thus scope for further research to explore the feasibility and acceptability of communicating results through mobile technology (phone calls and/or messaging applications) amongst this population. It remains unknown what waiting period would be acceptable and sufficient to retain most adolescents.

The perception of STI risk in this South African adolescent population was low despite reporting inconsistent condom use and half subsequently testing positive for at least one STI. Additionally, despite the high HIV burden in SA, nearly a third of YC participants declined a HIV test. It is possible that the perceived increased stigma of HIV over other STIs may have played a role. Given that adolescents appeared to be more enthusiastic about STI testing however, STI testing might be a useful hook to engage adolescents in SRH services generally.

The cost analysis unsurprisingly showed syndromic management to be the least costly option, as although it requires trained personnel, it requires minimal supplies, equipment, and medication. It is possible that the cost saving is offset by the poor performance of syndromic management, although this requires future assessment. This pilot study is one of the few POC STI testing implementation studies conducted in lower-income countries in an adolescent population. At least two African studies have found that specific POC diagnostics reduced over-treatment with antibiotics [31, 32]. Although POC technology used (GeneXpert) was more costly than syndromic management, and required extra space and electricity that may not be available in all settings, in countries that have already invested in GeneXpert laboratory-based testing platforms for TB,there may be an opportunity to integrate STI testing at lower cost.

This pilot study conducted under pragmatic conditions had some limitations. The study included a convenience sample of young people attending two well established adolescent SRH service distribution points known to be adolescent focused and friendly, which may not be generalisable to other primary care settings in South Africa. However, this did provide important feedback on whether POC diagnostic testing would be well received and how adolescents would view the waiting period. Although contact slips were given to participants testing positive for CT and/or NG, it is unknown whether disclosure took place or partners were successfully treated, and therefore re-infection may have occurred. This is the current standard of practice which is known to have limited efficacy [33, 34].

WHO has recommended aetiological testing as part of its 2016–2021 strategy for global STI control particularly among key populations [1]. The added cost of aetiological testing may be offset given the costs of managing the sequelae of untreated STIs, cost and harm of overtreatment with antibiotics and potential increased HIV risk. Larger implementation studies in LMICs should evaluate the feasibility of integrating STI test-and-treat programmes into existing HIV/SRH services. In particular, there is a need for research to understand how to integrate existing technologies into the most high-burden, vulnerable settings as well as to identify acceptable methods to support rapid results delivery and efficient targeted treatment for both patient and partner.

## Conclusions

STIs are highly prevalent amongst young people in southern Africa, with potential significant sequelae, A robust public health approach is required to address this, including increased resource allocation towards the integration of aetiological testing and targeted treatment of STIs into adolescent-focused health services.

### Supplementary Information

**Additional file 1.**

## Data Availability

The datasets generated and/or analysed during the current study are available in the University of Cape Town’s ZivaHub repository, https://zivahub.uct.ac.za/projects/The_STAX_Study/151755

## Abbreviations

CAB: Community advisory board
CT: *Chlamydia trachomatis*
HIV: Human immunodeficiency virus
HREC: Human research ethics committee
IQR: Inter-quartile range
LMICs: Low to middle income countries
MC: Mobile clinic
NAAT: Nucleic acid amplification test
NG: *Neisseria gonorrhoeae*
POC: Point of care
PrEP: Pre-exposure prophylaxis
SA: South Africa
SM: Syndromic management
SRH: Sexual and reproductive health
SSA: Sub Saharan Africa
STIs: Sexually transmitted infections
TB: Tuberculosis
UCT: University of Cape Town
WHO: World Health Organisation
YC: Youth clinic
YP: Young people
ZAR: South African rand
