# Supplementary material for: Acceptability, feasibility and cost of point of care testing for sexually transmitted infections among South African adolescents where syndromic management is standard of care
Source: BMC Health Serv Res. 2023 Oct 10;23:1078. doi: 10.1186/s12913-023-10068-8 (PMC10566254; doi:10.1186/s12913-023-10068-8)
Supplement: Supplementary file 1 — Additional file 1. [file 12913_2023_10068_MOESM1_ESM.pdf]

SEXUAL RISK BEHAVIOUR and SYMPTOM QUESTIONNAIRE

SITE	PARTICIPANT ID	VISIT DATE *(DD/MON/YY)*
______________________________	____________________	___ ___/ ___ ___ ___/ ___ ___

PLEASE CIRCLE THE PARTICIPANT'S RESPONSE

1. Have you had penile-vaginal or penile-anal sex in the last two years? (By penile-vaginal sex we mean when a man inserts his penis into his partner's vagina; penile-anal sex is when a man inserts his penis into his partner's anus)
Yes No

2. Are you having sex with anyone regularly?
Yes No

IF NO TO BOTH 1 & 2, PARTICIPANT IS NOT ELIGIBLE FOR STUDY

3. What was your reason for attending today? (more than one can be circled)
STI symptoms STI testing HIV testing Family planning/Contraception Other

4. How many people have you had either penile-vaginal or penile-anal sex with in the last 3 months?:
   a. One partner
   b. 1-5 partners
   c. More than 5 partners

5. How old were you when you first had penile-vaginal sex? _______

6. How often have you used condoms (male or female condoms) when you had penile-vaginal or penile-anal sex in the past 3 months?
1---------------2---------------3---------------4---------------5
Never Less than half the time Half the time More than half the time Always

7. Have you had more than one sexual partner in the past three months? (Multiple partners)
1---------------2---------------3---------------4---------------5
No Don't think so Not sure I think so Yes

8. Has/ have your partner/s had more than one sexual partner in the past three months (Multiple partners)
1---------------2---------------3---------------4---------------5
No Don't think so Not sure I think so Yes

9. Have you had sex with a person who is 5 years or more older than you in the past three months? (Intergenerational sex)
1---------------2---------------3---------------4---------------5
10. No Don't think so Not sure I think so Yes

11. Have your current sex partners tested for HIV? (Unknown partner status)
1---------------2---------------3---------------4---------------5
No Don't think so Not sure I think so Yes

12. Are any of your sexual partners HIV positive? (Discordance)
    1----------------2----------------3----------------4----------------5
    No   Don't think so   Not sure   I think so   Yes

13. Have you had any type of sex with someone for food, airtime, money, clothes, a place to stay etc in the past three months? (Transactional sex)
    1----------------2----------------3----------------4----------------5
    No   Don't think so   Not sure   I think so   Yes

14. Have you or your partner/s had an STI since your last visit? (STIs)
    1----------------2----------------3----------------4----------------5
    No   Don't think so   Not sure   I think so   Yes

15. How much do you think you are at risk of getting an STI?
    0% 10 20 30 40 50 60 70 80 90 100%

16. Do you have any of the following symptoms?

**Symptoms**	**Details**
Pain on urination (dysuria)	
Abnormal vaginal discharge	
Pain during sex (dyspareunia)	
Unusual vaginal odour	
Genital irritation	
Pain in lower abdomen	
Warts or ulcers or sores	
Bleeding after sex	
Genital rash	
Swollen inguinal lymph nodes	

This is the end of the sexual risk behaviour and symptom survey for participants. Thank you for your time and participation in the survey. Please feel free to ask questions you may have.

Date: ___ ___ / ___ ___ ___ / ___ ___   **Staff member initials:**__________________
